# The Journal, 1899–1900

**Published:** 1899-12

**Authors:** 


### EDITORIAL.

### THE JOURNAL, 1899–1900.

Every dark cloud is said to have its silver lining, and nothing could be truer than this adage applied to the veterinary world. After four or five years of decreasing values in the equine markets; after the introduction of the bicycle, with the cheaper attraction that served well its purpose for a time in view of general business depression; then the fitful assault upon the equine species by the much vaunted horseless age, and the still-to-come auto-trucks and auto-wagons, we have just felt the returning tide of prosperity during the past few months from the daily increasing value of horses all over our land. The scarcity of good horses for every kind of service has made the price advance, and with an increasing daily demand and a less than normal supply these conditions have again stimulated breeding, made horse centres active, buyers plenty, and the care and keeping of these pleasure givers, health restorers, and business promoters a matter of such moment as to again call into service the veterinarian in every village, town, and city.

Increased attention to veterinary sanitary subjects, broader and better meat-inspection, keener and more thorough milk-inspection, and veterinary sanitary police work have all taken fresh impetus and added to the joy and welfare of the profession.

The Journal has enjoyed the past year the best since 1894, and in thanking our readers for their kind support, and begging indulgence in that our issues have not always been on time, we assure them all that for 1900 we will again resume our wonted regularity and send forth our numbers better and more valuable than ever before. We extend to all our readers and the profession our sincere wishes that 1900 will prove an old-time period of prosperity to all.

